# Saudi Arabia’s Healthy Food Strategy: Progress & Hurdles in the 2030 Road

**DOI:** 10.3390/nu13072130

**Published:** 2021-06-22

**Authors:** Faisal Fahad Bin Sunaid, Ayoub Al-Jawaldeh, Meshal Wasel Almutairi, Rawan Abdulaziz Alobaid, Tagreed Mohammad Alfuraih, Faisal Naser Bensaidan, Atheer Shayea Alragea, Lulu Ali Almutairi, Ali F. Duhaim, Talal Ali Alsaloom, Jana Jabbour

**Affiliations:** 1 Healthy Food Department, Saudi Food and Drug Authority, Riyadh 13513-7148, Saudi Arabia; ffsunaid@sfda.gov.sa (F.F.B.S.); mwmotairi@sfda.gov.sa (M.W.A.); raobaid@sfda.gov.sa (R.A.A.); tmfuraih@sfda.gov.sa (T.M.A.); fnbensaidan@sfda.gov.sa (F.N.B.); asraqea@sfda.gov.sa (A.S.A.); lamutairi@sfda.gov.sa (L.A.A.); afduhaim@sfda.gov.sa (A.F.D.); tasalloom@sfda.gov.sa (T.A.A.); 2 Regional Office for the Eastern Mediterranean (EMRO), World Health Organization (WHO), Cairo 11371, Egypt; aljawaldeha@who.int; 3 Nutrition Department, School of Health Sciences, Modern University of Business and Sciences, Beirut 113-7501, Lebanon

**Keywords:** health policies, nutrition, nutrient labels, trans fatty acids, non-communicable diseases, obesity, KSA, Eastern Mediterranean region

## Abstract

The Kingdom of Saudi Arabia (KSA) is a leading country worldwide in the prevalence of non-communicable diseases (NCDs), which alone can explain 73% of mortality in the country. In response to the heavy burden of NCDs, the Saudi Food and Drug Authority (SFDA), in collaboration with other government entities, developed a healthy food strategy (HFS) aimed at enhancing healthy lifestyles and reducing the intake of salt, sugar, saturated fatty acids (SSF) and trans fatty acids (TFA). The objectives of the HFS, to facilitate consumers’ identification of SSF and reduce the SSF and TFA content in food items, were addressed in collaboration with key stakeholders in the public and private sectors of the food industry. These reforms included voluntary and mandatory schemes to display nutrition information in food and beverage establishments, display allergens on food menus, encourage the adoption of front of pack nutrient labels (FoPNLs) on food products, ban the use of partially hydrogenated oils and establish limits for sodium composition in breads and selected food products. This manuscript contextualizes the HFS and presents the results of monitoring initiatives undertaken by the SFDA to assess compliance with these reforms.

## 1. Introduction

With the unparalleled rise in non-communicable diseases (NCDs) in the twenty-first century, efforts have been put towards generating dietary guidelines, promoting health counseling and enhancing consumers’ personal accountability [1]. With time, health care and governmental agencies have identified the multifactorial social determinants of obesity, which also involve legal reforms, industrial manufacturing and preparation in food and beverage establishments [2]. Recognizing their association with an increased risk of cancer, cardiovascular diseases, and overall mortality, sodium, added sugars, saturated fatty acids (SSF) and trans fatty acids (TFA) have been the main nutrients targeted in such policies and interventions [3,4,5,6]. The focus has been on increasing consumers’ awareness of their health hazards, facilitating their identification by consumers and reducing their content in food items [2]. The United Kingdom (UK) set an example with an interdisciplinary multicomponent initiative to be followed with a salt-reduction program [7]. This strategy included awareness campaigns for the public, as well as legal reforms with the industry and food establishments, which ensured gradual and sustainable reductions in sodium content. Despite the trends of increasing sodium consumption worldwide, this program succeeded in reducing sodium intake in the UK by 15% over 7 years [7].

The Kingdom of Saudi Arabia (KSA), part of the Eastern Mediterranean region (EMR), is one of the fastest growing economies in the world. The escalation in household income in the beginning of the 21st century was accompanied with a fast nutrition transition and an increased consumption of SSF, associated with a heavy burden of obesity, food allergies, and NCD [8,9,10,11]. Recent data by the World Health Organization (WHO) and the Global Burden of Disease (GBD) revealed that KSA is among the top countries in the region and the world in the prevalence of obesity and diabetes. NCDs explain 73% of mortality and kill more than 900,000 individuals yearly, and high body mass index (BMI), prevalent in more than 65% of the population, is the leading factor of years lived with disabilities (YLD) in KSA [12,13,14,15,16,17]. Among children and adolescents, rates of overweight and obesity have also increased, exceeding 31% in this subpopulation [18,19,20,21,22,23]. NCDs have been estimated to burden the country, on an annual basis, with 19 billion United States dollars (USD) in direct costs and 13 billion USD in indirect costs from lost productivity [24]. In response to the heavy burden of obesity and NCDs, the KSA’s government developed nutritional guidelines and strategies, the last of which was the healthy food strategy (HFS) established by the Saudi Food and Drug Authority (SFDA), as part of Saudi Vision 2030 [25]. The HFS, planned in September 2017 and officially launched in September 2018, incorporated a series of nutritional reforms and educational campaigns. This article aims to provide readers with an overview of the HFS reforms and the results of the monitoring initiatives. It is structured as follows: Section 2 describes nutrition reforms implemented prior to the HFS in the KSA; Section 3 presents a summary of nutrition policies implemented as part of the HFS; Section 4 reviews monitoring initiatives lead by the SFDA, and Section 5 discusses the HFS by comparing this study’s findings to the literature on the subject.

## 2. Review of Nutrition Reforms Prior to the HFS Implementation

In 2012, the Ministry of Health established the Healthy Food Palm Dietary Guidelines to provide culturally relevant guidance to health care providers and individuals on healthy diets [26]. These food-based dietary guidelines, meant to be employed by health care providers and to be incorporated in awareness campaigns and schools’ curricula, provided culturally relevant guidance on the recommended daily food-group intake by age category [26].

In KSA, all edible products need to comply with the guidelines of the Gulf Standardization Organization (GSO) and the SFDA [27]. The GSO issues two forms of governing documents: technical regulations that substitute for national policies and standards, the adoption of which is optional for member nations [28]. In 2013, the SFDA enforced general labeling requirements on prepackaged food items, exported and locally produced, available in the country’s market. Products needed to have a comprehensive list of ingredients written in descending order in Arabic [28]. In 2018, the SFDA, in line with the GSO’s technical regulation, imposed an updated nutritional-labeling requirement on all prepackaged food and beverages available in the Saudi market [28]. This regulation enforced the display of all macronutrients, including total fat, saturated fat, total carbohydrates, total sugar and sodium, as well as selected vitamins. This regulation excluded items that had negligible amounts of macronutrients, SSF and TFA, such as spices, small packages, fresh fruits and vegetables, as well as packages that had single nutrients and/or ingredients. Fresh and chilled meats, poultry and fish were also excluded from this regulation as well as products sold to consumers from preparation points (e.g., in restaurants and coffee shops) [28]. Recognizing the health hazards of TFA consumption, the SFDA sat an upper limits for TFA of 2% in fats and oil products and 5% for other products in 2015 [29]. A grace period was provided for manufacturers to adapt the composition of food products until 2017, when the TFA limits were enforced as a regulation. Finally, because KSA residents were identified as the fifth largest consumers of sugar-sweetened beverages (SSB) in the world, a flat sin tax was imposed by the General Authority of Zakat, Tax and Customs (GZAT) on carbonated and energy drinks in 2017 [30,31]. The latter reform lead to a reduction in sales of carbonated beverages by around 33% compared to untaxed beverages in KSA [32]. All in all, cross-sectional studies revealed that despite the implementation of several reforms and the successful reduction in carbonated drinks consumption, the gap remained large between dietary recommendations and goals in KSA. Less than 10% of assessed individuals met the recommendations for fruit and vegetable intake; less than 50% complied with the guidelines for fish consumption in 2013, and salt intake was 9.3 g/day in 2016, almost double the relevant WHO recommendation of 5 g/day [11,33].

## 3. The HFS: Description of the Health Reforms

In 2017, the SFDA moved from setting individual regulations to conceptualizing the HFS, a wider strategy that aimed at improving nutritional and health indicators. While the SFDA took the lead in establishing the whole strategy, it collaborated with key governmental entities (e.g., the ministry of health, ministry of municipality, ministry of environment, water and agriculture, as well as the key ruler) and universities and ensured the buy-in of private entities such as food manufacturers and establishments. Components of HFS reforms included the elimination of industrial TFA and the reduction of the SSF content in food items, the empowerment and education of consumers and the enhancement of public awareness in partnership with food establishments and the food industry. Our research team reviewed HFS-related policies and surveillance reports published by the SFDA. Table 1 and the following section summarize the voluntary and mandatory schemes that were established under the HFS umbrella. The majority of regulations provided a grace period for collaborators between issuance and enforcement. During this time, the SFDA generated guidelines that were relevant to the regulations, implemented educational interventions to raise awareness among consumers and provided workshops for collaborators to discuss and clarify the regulations.

### 3.1. Display of Nutrition Information and Regulation of Juice Compositions in Food and Beverage Establishments

To empower individuals to make healthy decisions when eating out, the SFDA invited all food establishments in KSA (restaurants, hotels and coffee shops) in 2017 to display caloric information on their menus and to have their meals’ nutrient compositions available for each item in the establishment to answer customers’ inquiries [34]. To make caloric information relevant to the consumers, menus also included the recommended caloric intake for adults and children. This voluntary approach, which progressed to a mandatory scheme in 2019, provided several options to food establishments to calculate the caloric content of the offered items: analysis in a certified food laboratory, use of a list of approved mobile applications and/or consultation with a licensed dietitian [34]. The display of caloric content was the legislation that required the largest number of workshops across the KSA, because the food establishments found it challenging to calculate the nutrient composition of their meals. During these workshops, the food industry representatives expressed concern over the cost of the analysis of meals in laboratories. The SFDA, in its turn, developed a free website facilitating the calculation of nutrient composition [35]. The display of food allergens on food establishment menus, enforced in the same year, aimed to empower consumers suffering from food allergies to make healthy choices and to prevent allergic reactions. This regulation, enforced in 2019, required food establishments to list 14 common food allergens on their menus [36]. The SFDA also targeted juice shops and outlets with a regulation in 2020 that mandated the display of nutrient labels on juice products. This regulation controlled the preparation and nutrient composition of added sugars in fresh juices, nectars and fruit drinks [37] (Table 1).

### 3.2. Front of Pack and Back of Pack Nutrition Labelling

The difficulty of comprehending information on nutrition labels led many governmental agencies to adopt different types of labels allowing consumers to make quick and informed decisions while shopping [38]. In 2018, the SFDA invited local and international manufacturers to join a voluntary pledge to apply front of pack nutrition labels (FoPNL) on prepackaged foods [39,40]. The recommended FoPNL featured the multiple traffic light (MTL), which uses red, amber and green colors to reflect elevated, moderate and low composition of selected nutrients, respectively [41]. MTL was used for SSF and total fat content expressed per 100 g or per 100 mL. On the same topic of nutrient labeling, and as previous policies did not mandate the display of TFA, added sugar, total cholesterol and fiber on food products’ nutrition information [28], a policy enforcing their display on the back of pack label on all food manufacturers was issued as part of the HFS [42]. During the grace period, when discussing the reforms with the SFDA, food manufacturers were concerned about the lack of time to analyze and display the additional nutrients. The SFDA hence provided several extensions before enforcing this legislation.

### 3.3. Trans Fatty Acid Regulations

Following up on the TFA limits enforced in 2017, the SFDA decided to ban all partially hydrogenated oils (PHO) for use in food manufacturing. The SFDA signed a voluntary agreement with representatives of the food industry in 2018 [29]. The SFDA supported the food industry by increasing the supply of healthy fats and oils in the market and by conducting workshops to educate manufacturers on strategies to replace PHO with healthier alternatives. In 2020, with the enforcement of this regulation, the KSA became the first country in the EMR to have a “best practice TFA policy” that imposed the elimination of industrially produced TFA in food products [6].

### 3.4. Restricting Sodium Content in Food Items

In 2019, with the aim of reducing sodium content in food items, the SFDA enforced a limit of 1 g/100 g for breads and for ayran (a traditional yogurt drink rich in salt) on manufacturers [43,44]. This regulation was followed in 2019 with an invite from the SFDA to food manufacturers to abide by limits for sodium content for 22 processed food items, inspired by limits set by Public Health England [45,46]. During educational workshops, small-scale bakeries expressed concerns on how to modify the content of breads without affecting their stability. SFDA supported these manufacturers by providing them with relevant recipes.

### 3.5. Sin Tax on all SSB

After the implementation of a flat tax on carbonated and power beverages, the GZAT, in collaboration with SFDA and under the umbrella of the HFS, extended the tax to include all SSB including flavored sweetened milk, juices, etc. [47]. The flat tax, enforced in 2019, involved a 50% tax on all SSB and carbonated beverages compared to a 100% tax on energy drinks.

### 3.6. Educational Campaigns

Several educational campaigns were launched by the SFDA, as part of the HFS, to promote healthy diets among consumers. Some of these campaigns were implemented in the workplace. The first initiative targeted food establishments in governmental and non-governmental agencies to increase fiber intake and to reduce SSF and TFA intake in the served meals and beverages. This campaign, initiated in 2019, involved a ban on the sale of carbonated beverages and the use of palm and coconut oil in food preparation, encouraged healthy cooking methods, replaced juice drinks with fresh drinks and included labels on sugar and salt packets with the WHO-recommended daily limits for the establishments adopting it. It also encouraged the wider availability of fiber-rich food sources and low-salt alternatives and the inclusion of healthy alternatives for sweets in their menus. Corporate food establishments across the KSA interested in this initiative were invited to sign up with the SFDA and implement it in their corporate restaurants and coffee shops. Another initiative in the workplace was recently launched as a collaboration between the Food and Agriculture Organization (FAO) and the SFDA to celebrate the fruit and vegetable (F&V) year [48]. This initiative, launched in April 2021, aimed at increasing the awareness of the health benefits of F&V in the workplace and the importance of limiting their waste. It involved sharing nutrition-education material and infographics via email and on screens in the firms. It also promoted the distribution of F&V to the offices and making them available in the areas where breaks are taken. This campaign encouraged corporate food establishments to include more F&V options on their menus, incorporate the logo for the international year for F&V on products produced in the food establishments, such as coffee cups and juices. Funding for these campaigns had to be done by the firms themselves.

Another campaign focused on food establishments providing delivery services. It incorporated a limit for calories, sodium, added sugar and the total fat composition of meals, as well as a reduction in portion sizes. This campaign targeted consumers by designing and distributing a guide for healthy meals allowing them to assess how healthy a meal is by comparing its composition to the WHO recommendations. Establishments that had meals that abided by the set limits were granted the privilege by the SFDA to label the meal as a “Balanced Meal”. In addition to these campaigns, the SFDA lead several initiatives to educate the public on healthy-eating habits via live events and through their social media networks.

**Table 1:** Regulations and pledges of the Healthy Food Strategy.

Policy Description and Reference	Type of Policies and Relevant Dates of Enactment and/or Enforcement	Key Collaborators	Policy Goal
Display of caloric information on food and beverage products in menus of food establishments (restaurants, hotels and coffee shops). SFDA.FD 20 [34]	Voluntary pledge in 2017 Enforced in 2019	Food establishments (restaurants, hotels and coffee shop companies)	Empower individuals to make healthy decisions when eating out.
Declaration of allergens on food establishments’ menus. SFDA.FD 56 [36]	Issued in 2018 Enforced in 2019	Food establishments (restaurants, hotels and coffee shops)	
Regulation of nutrient composition in fresh juices, nectars and fruit drinks and their display on products. SFDA.FD 5001 [37]	Issued in 2019 Enforced in 2020	Food establishments (restaurants, hotels, coffee shops, juice shops and supermarkets that sell juices)	Encourage food and beverage establishments to withhold adding sugars to juices. Reduce consumption of sugars and empower individuals to make healthy choices while eating out.
Display of front of pack nutrition labels on prepackaged foods for sugar, sodium, saturated fat and total fat. Encourage the use of multiple traffic light labeling, a graphical illustration allowing individuals to understand nutrient composition from a color code. SFDA.FD 42 [40]	Voluntary scheme in 2018	Food manufacturers	Encourage manufacturers to display nutrient content in a comprehensible way to consumers. Empower individuals to make healthy choices while shopping. Reduce consumption of added sugars.
Mandate for back of pack nutritional labels that display the added sugar, total cholesterol, fiber and TFA on prepackaged food items. SFDA.FD 2233 [42]	Issued in 2018 Enforced in 2021	Food manufacturers	
Ban of all partially hydrogenated oils (PHO) in food manufacturing. SFDA.FD 2483 [29]	Issued in 2018 Enforced in 2020	Food manufacturers	Encourage manufacturers to reformulate their products to reduce industrially produced TFA content. Reduce consumption of industrial TFA.
Mandate for a sodium limit for breads and (1 g/100 g) and ayran (yogurt drink) (1 g/100 g). SFDA.FD 2362 [43] SFDA.FD 57 [44]	Issued in 2018 Enforced in 2019	Food manufacturers	Collaborate with manufacturers to reformulate their products to reduce sodium composition. Reduce sodium consumption.
Recommendation of a sodium limit for 22 processed food categories: cheeses, butter, fat spreads, beans, cooked and canned soups, ready meals, pizza, cakes, table sauces, biscuits, pasta, canned fish, meats, canned vegetables, chips, cooking sauces, potatoes, flavor enhancers and beverage powders. SFDA.FD 59 [46]	Voluntary scheme in 2018	Food manufacturers	
Enforcement of a flat tax of 50% on all sugar-sweetened beverages, including carbonated drinks, juices, and dairy products [47].	Enforced in 2019	General Authority of Zakat, Tax and Customs	Reduce sugar-sweetened beverages consumption.

TFA: Trans fatty acids.

## 4. Assessment of the HFS Acceptance and Implementation

To monitor the compliance rates with the HFS reforms, the SFDA conducted a series of surveillance campaigns. These surveillances were either conducted in the city of Riyadh, the capital of the KSA, to provide preliminary insight or at the national level across five regions in the kingdom. Non-compliance with the mandatory regulations was treated as a legal violation by the SFDA. As for the non-mandatory, voluntary pledges, poor compliance was addressed by the SFDA with encouragement to comply with recommendations.

### 4.1. Assessment of Nutrient and Allergens Information in Food Establishments

National inspection campaigns monitored the compliance of food establishments with the HFS regulations. The first campaign conducted in 2019, targeted food establishments (restaurants, coffee shops, ice cream shops, fresh juice shops, corporate cafeterias and bakeries), assessing if they calculated nutrients and displayed calories on their menus. Inclusion criteria included food outlets with five or more branches in KSA. Data collected on 1363 establishments revealed that 27% did not display caloric content; 25% had incomplete display of caloric content, and 19% had incorrect calculation. A similar national campaign conducted in the same year assessed the compliance of food establishments with the display of food-allergen information. Findings revealed that 37% were not compliant with declaring food allergens on their menus. Finally, surveillance assessing restaurants, juice and coffee shops’ compliance with the juices regulation was conducted in 2020. Results showed that 60% of establishments did not display calories on the food menus and/or used sweeteners in the preparation of fresh juices.

### 4.2. Front of Pack Nutrition Labels Evaluation

A year after inviting manufacturers from the private sector to join the voluntary pledge to include FoPNL on their products, the SFDA assessed products for the availability and the type of FoPNL employed. In the period extending from March 2019 until March 2020, the SFDA surveilled food products through a secondary data analysis of a dataset that it managed with information and pictures on food and beverage products available in the Saudi market. Products that included FoPNL were included. Exclusion criteria included product duplicates and those that had different flavor and/or size. Data were collected on the name and brand of the product, food category, type of FoPNL employed, manufacturing company type (local or international) and nutrient composition (SSF content). Products from 4335 companies were screened and 80 companies (1.8%) had FoPNL on 119 unique products. Beverages were most likely to have FoPNL (30% of analyzed products), followed by dairy products (23%) and confectionaries (13%) (Supplementary Table S1). Most companies employed Guidelines Daily Allowance (63%), followed by MTL (36%), and a Health Star Rating (1%). It is worth noting that the majority of products that displayed MTL had a low content of salt, saturated fat and total fat. The sugar content of these products was mostly moderate (40% of products) and elevated (42% of these products). Most of the products using MTL (65%) were compliant to the SFDA recommendation of displaying the FoPNL per 100 g or mL.

### 4.3. Surveillance of the Sodium Content in Breads and Food Products

The SFDA conducted national surveillance in 2019 after the regulation on sodium limits in breads was enforced. It involved an assessment of the nutrition label of bread products in bread factories, automatic bakeries and semi-automatic bakeries. The results revealed that, out of the 297 products assessed, 85% were compliant with the advised sodium limit of 1%.

**Figure 1:**
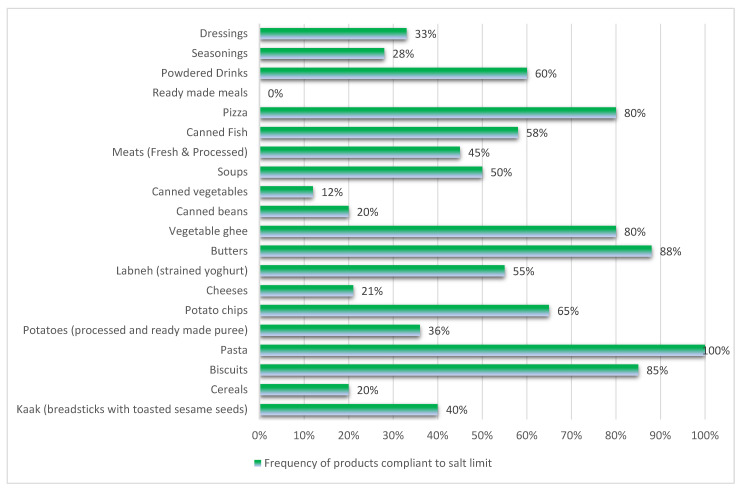
Frequency of products compliant with the salt limit.

To monitor the compliance of food producers with the salt-limit recommendations set by the SFDA, a surveillance of food products available in supermarkets was conducted in the city of Riyadh in October 2020. Compliance was evaluated by comparing the products’ sodium composition from the nutrition labels in three supermarkets. These supermarkets were selected as they were the most visited supermarkets in the capital. Products (*n* = 267) were assessed, and 261 were included from 21 food categories, with 47% found to be compliant with the relevant SFDA recommendations. Compliance ranged from 0% for ready-made meals to 100% for pasta products. Kaak (breadsticks with toasted sesame seeds), cereals, potatoes, cheeses, canned beans and vegetables, meats (fresh and processed), ready-made meals, seasonings and flavorings had more than 50% of their products exceed the sodium limit sat by the SFDA (Figure 1). There were variations in compliance rates within some groups. For example, within the meat group, all fresh ground meat items were compliant with the sodium limits, whereas 25–50% of pastries prepared with meats, hamburgers and all of the processed meats were non-compliant with the SFDA sodium recommendations. Within the labneh group, 80% and 25% of full-fat and low-fat labneh products, respectively, were compliant with the sodium limits.

### 4.4. Surveillance of SSF in Children Food Products

**Figure 2:**
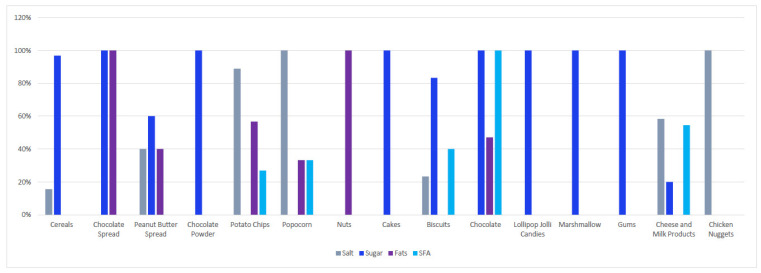
Percentage of children’s food products with salt, sugar, total fat and saturated fatty acid composition exceeding WHO limits. SFA: saturated fatty acids.

A surveillance campaign in supermarkets in KSA’s capital, Riyadh, assessed the compliance of food and beverage products targeting children with SSF guidelines. Supermarkets (*n* = 3) were selected based on their popularity, using the same criteria as the sodium-surveillance campaigns. Products that included toys and/or that featured the picture of a cartoon character were included in the surveillance campaign. Exclusion criteria included products for which consumption is advised by health authorities and those known to be low in SSF (e.g., unflavored milk). Nutrient composition of the included products was compared to the WHO guidelines (limits: 10% for saturated fats and added sugar, 30% for total fat, 1 g/100 g for salt). From the screened items (*n* = 294), 18 products were excluded because they had incomplete nutrition information. All products had a cartoon character featured, and 6% had toys included with the products. Of the included products, 91% had at least one nutrient that exceeded the recommended limits. Figure 2 showcases the percentage of products exceeding WHO limits for SSF and total fat by food category. All assessed products in the sweets and cereals categories exceeded the sugar limit. Similarly, all products in the chicken nugget, potato chip, and popcorn groups exceeded the salt limit (Figure 2).

### 4.5. Surveillance of TFA in Food Items

In July 2020, a national inspection campaign was conducted by the SFDA to assess the compliance of food manufacturers with the TFA regulations. Assessment was done by reviewing the manufacturing techniques and the ingredients to evaluate their PHO content. In case of inconsistency between manufacturing techniques and nutrient labels, laboratory analysis of food products was conducted through high-performance liquid chromatography (HPLC). Out of the 1117 manufacturers evaluated, 37% were local manufacturers, and 7% had non-compliant products. Of the assessed items, 20% were non-compliant because they had TFA containing ingredients that were not mentioned in the product information and/or because the nutrient labels did not reflect the correct nutritional analysis.

## 5. Discussion

The HFS is a comprehensive strategy aiming at reducing SSF and TFA composition in food items and empowering consumers to make healthy choices while shopping and eating out and in. This manuscript provided an overview of the HFS’ nutrition policies and recommendations implemented in the KSA and offered insight on the level of compliance with these reforms and on the challenges faced by stakeholders.

Despite the successful launch and implementation of the HFS, compliance with the regulations can be considered moderate a few years afterwards. While some regulations had an elevated compliance rate (e.g., TFA regulation, sodium limit in breads, display of food allergens), others did not (e.g., preparation and display of nutrition labels on juices, compliance of children’s food items with the SSF limits, adoption of FoPNL). Only 2% of assessed food manufacturers adopted the FoPNL, the majority of which had a moderate content of salt, saturated fat and total fat. The rest of the policies had a fluctuation in compliance rate depending on the evaluated categories (e.g., reform on the sodium content of processed products).

To assess the effect of the HFS on product composition, comparisons were made between assessments that preceded the HFS implementation and those succeeding it (presented in our study). A cross-sectional study assessing the presence of TFA-containing products in the Saudi market, conducted in 2014–2016, revealed that 31% of the evaluated products contained TFA, and only 20% listed TFA in the nutrient label [49]. Our study, based on SFDA surveillance conducted in 2020, showed marked improvement in TFA labeling, with 80% of the assessed products displaying TFA information as recommended by the reforms. Moreover, a cross-sectional study evaluating food products’ nutrient labels and compliance with the SSF SFDA recommendations in 2016–2017 revealed that more than 20% of evaluated products did not comply with the sodium, sugar and total-fat limits [50]. Our analysis yielded similar findings. Among pediatric products, all sweet and cereal products exceeded the sugar limit, and all products in the chicken nugget, potato chip and popcorn groups exceeded the sodium limits. The products targeting all age categories showed fluctuating compliance with the sodium regulation across food categories. All in all, there was a marked improvement in TFA labeling. Yet, no significant improvements in SSF composition have been observed so far, and manufacturers seemed to be reluctant to display FoPNL for products that exceed the SSF limits. These findings highlight the need to support food manufacturers in reformulating their products and in designing FoPNL, as a gap still exists between the HFS recommendation and actual practice.

The need to design interventions to enhance the knowledge and behavior of the general population and to support restaurant owners with nutrition and food-science skills to improve the composition of their meals was also identified. A recent cross-sectional study assessed the knowledge, attitude and behavior of restaurant owners and consumers towards the energy labeling policy in food establishments. Participants’ knowledge of daily caloric requirements for inactive adults was low. Even though the consumption of low and high calorie meals respectively increased and decreased by 44% and 39%, 51% of subjects were less likely to eat at restaurants displaying energy content. Among restaurant owners, half of them did not know the reason behind the enforcement of this policy, and most were resistant to modifying their recipes to reduce SSF content [51]. At the consumer end, several cross-sectional studies conducted between 2019 and 2020 among adults and university students assessed their knowledge, attitude and behavior towards several dietary components, including SSF. The results revealed that participants had poor knowledge and an elevated consumption of salt, good knowledge of added sugar health risks, yet an elevated intake of sweets and fast foods and a low consumption of F&V [52,53,54]. An assessment among Saudi adults prior to the HFS implementation related poor fiber intake to the expensive prices of fruits and vegetables, limited availability and dislike of their taste [55]. Through educational campaigns, the SFDA intended to address the availability of F&V in the workplace. Yet, in view of a lack of analysis of the uptake and compliance with these campaigns, we are unable to assess the success rate of this component of the HFS.

At the regional level, KSA was the first country to have a “best practice TFA policy” and to implement a flat tax on carbonated beverages and energy drinks [6,56]. It was among the first to implement a HFS and encourage the use of FoPNL, along with Iran and the United Arab Emirates (UAE), and to enforce limits for sodium content in breads, along with the occupied territories of Palestine, Oman and the UAE [56,57]. Despite the early adoption of the FoPNL pledge in KSA, a minimal number of products have used it, compared to Iran, who was the first to mandate it in the region (2% vs. 80%, respectively) [58]. Yet, the accuracy of the FoPNL labels was found to be much lower in Iran for sweets compared to our study findings in KSA [59]. This latter finding justified the lack of trust of consumers in labels and having only 15% of the population rely on FoPNL in Iran to make shopping decisions [60].

This study has many strengths and limitations. Starting with the latter, the SFDA relied on extracting data from nutrition labels to monitor the compliance of food products, and laboratory analysis was not performed in all assessments due to the heavy financial cost incurred in such large surveillance studies. Moreover, the surveillance conducted had slight differences in methodology depending on the baseline data and assessed outcomes, with some applied on a national scale and others restricted to the city of Riyadh. Lastly, the SFDA did not collect data to analyze the interest, adoption and efficiency of the implemented educational campaigns. This study has many strengths, as it provides a comprehensive overview of policy changes in KSA using reliable data from the SFDA and presents findings of large surveillance surveys conducted shortly after the implementation of the HFS reforms.

## 6. Conclusions

This review provides a valuable resource to policymakers and stakeholders on the nutrition reforms in KSA. The HFS was introduced at a key time to alleviate the burden of NCDs on the residents’ quality of life, morbidity, mortality and occurrence of heavy healthcare costs. The HFS successfully addressed important components, such as food and beverage composition and nutrient labeling for manufactured products and those available in food establishments. The HFS’s positive influence was observed in the reduced TFA and sodium content of some products and the increased sales of low-calorie meals in food establishments. Yet, the HFS was neither able to improve the SSF content of many products nor able to encourage food manufacturers and restaurant owners to reformulate their products nor able to markedly influence consumers’ dietary patterns. SFDA is currently working on regulations and standards to encourage national and international food producers to innovate healthy food choices. Additional measures that would be instrumental include an assessment of the response to educational campaigns and of the changes in consumers’ and stakeholders’ knowledge, attitude and behavior towards the SSF components of the HFS.

## Data Availability

3rd Party Data Restrictions apply to the availability of these data. Data was obtained from the Saudi Food and Drug Authority and are available from the author Faisal Fahad Bin Sunaid with the permission of Saudi Food and Drug Authority.

## Supplementary Materials

The following are available online at https://www.mdpi.com/article/10.3390/nu13072130/s1, Table S1: Food category of products n (%) classified by type of FoPNL, Table S2: Nutrients based on MTL codes’ criteria.
