# Supplementary material for: Saudi Arabia’s Healthy Food Strategy: Progress & Hurdles in the 2030 Road
Source: Nutrients. 2021 Jun 22;13(7):2130. doi: 10.3390/nu13072130 (PMC8308336; doi:10.3390/nu13072130)
Supplement: Supplementary file 1 [file nutrients-13-02130-s001.zip › nutrients-1208657-supplementary.pdf]

Saudi Arabia's Healthy Food Strategy: Progress & Hurdles in the 2030 Road

Supplementary Material

Supplementary Table 1: Food category of products n (%) adopting FoPNL

**Categories**	**Multiple Traffic Lights (n=48)**	**Health Star Rating (n=1)**	**Guidelines Daily Allowance (n= 71)**
**Beverages**	17 (35.4)	0 (0)	19 (27.1)
**Cereals**	1 (2.1)	0 (0)	11 (15.7)
**Confectionary**	11 (22.9)	0 (0)	5 (7.1)
**Dairy Products**	10 (20.8)	1 (100)	17 (24.3)
**Sweet Spreads**	5 (10.4)	0 (0)	9 (12.9)
**Others**	4 (8.3)	0 (0)	9 (12.9)

FoPNL: Front of Pack Nutrient Label

Supplementary Table 1: Nutrients categorization based on the Multiple Traffic Light' criteria

**Categories**	**Green**	**Amber**	**Red**
**Sugar**	9 (19)	19 (40)	20 (42)
**Salt**	44 (92)	3 (5.8)	1 (1.9)
**Total Fat**	31 (65)	5 (10)	19 (27.1)
**Saturated Fat**	31 (65)	0 (0)	11 (15.7)

Green: Low level; Amber: Moderate level, Red: Elevated level
